# Supplementary material for: Fundamental limits of parasitoid-driven host population suppression: Implications for biological control
Source: PLoS One. 2023 Dec 22;18(12):e0295980. doi: 10.1371/journal.pone.0295980 (PMC10745201; doi:10.1371/journal.pone.0295980)

In[ ]:=
```
(*Egg Parasitoid *)
Clear [R, c, Carr, H, P, k]
c1 = (R - 1) / R / Carr;
Plot[{1 / R, c /. FindRoot[
     {((1 - ⅇ^-z) R (-ⅇ^z + R))/(-1 + R) == z / c, -1 + c ⅇ^z (ⅇ^z - R) + R == 0}, {z, Log[R] / 2}, {c, 2 / R}]},
 {R, 1.01, 10}, PlotRange → {{1.01, 10}, {0, 4}}, Filling → {1 → {2}},
 TicksStyle → {{FontSize → 20}, {FontSize → 15}},
 PlotStyle → Thickness[0.016], PlotStyle → {Black, Black}]
```

Out[ ]=

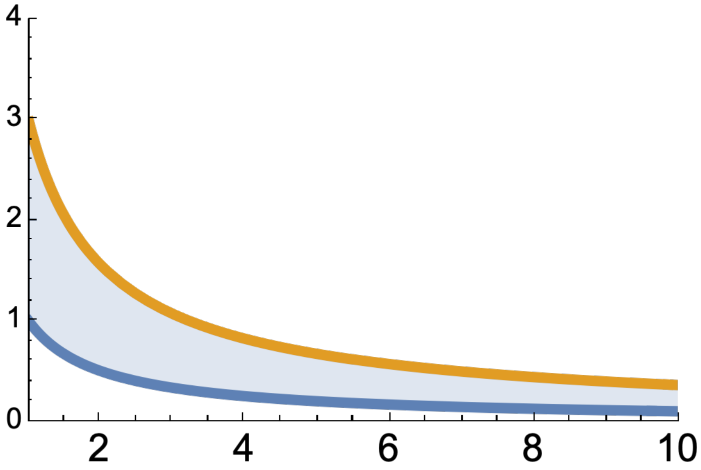

In[ ]:=
```
(*Pupal Parasitoid*)
Clear [R, c, Carr, H, P, k]
c1 = (R - 1) / R / Carr;
Plot[{1, c /. FindRoot[{- (e^-z (-1 + e^z) (e^z - R))/(-1 + R) == z / c, 1 + (c e^z (e^z - R))/((-1 + R) R) == 0},
    {z, 0.65 * Log[R]}, {c, 3}]}, {R, 1.1, 10}, PlotRange → {{1.2, 10}, {0, 4}},
  Filling → {1 → {2}}, TicksStyle → {{FontSize → 20}, {FontSize → 15}},
  PlotStyle → Thickness[0.016], PlotStyle → {Black, Black}]
```

Out[ ]=

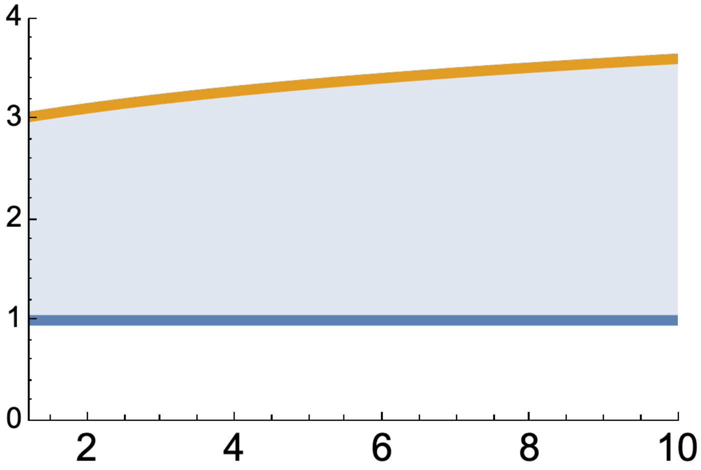


```
Clear [R, z, c]
Plot[{ ((Exp[z] - 1) (z + 1))/(R - 1) /. FindRoot[z + 1 == (R (Log[R] - z))/(R - Exp[z]), {z, 0.5`}],
  - (e^-z (e^z - R))/(-1 + R) /. FindRoot[{e^-2 z (-1 + e^z) R == z}, {z, Log[R]/2}],
  (-e^z + R)/(-1 + R) /. FindRoot[{e^-2 z (-1 + e^z) R == z}, {z, Log[R]/2}]}, {R, 1, 20}, PlotStyle →
   Directive[Hue[0.67`, 0.6`, 0.6`], Opacity[1.`], AbsoluteThickness[3.3`]],
  PlotRange → {{1, 20}, {0, 0.7}}, TicksStyle → {{FontSize → 20}, {FontSize → 15}},
  Ticks → {{1, 5, 10, 15, 20}, {0, 0.1, 0.2, 0.3, 0.4, 0.5, 0.6, 0.7}}]
```

In[∘]:=
```
(*Larval Parasitoid *)
Clear [R]
Carr = 1;
c1 = (R - 1) / R / Carr;
Plot[{c1 / Log[R], c1 / z /. FindRoot[z + 1 == R * (Log[R] - z) / (R - Exp[z]), {z, 0.5}]},
 {R, 1.05, 10}, PlotRange → {{1.05, 10}, {0, 4}}, Filling → {1 → {2}},
 TicksStyle → {{FontSize → 20}, {FontSize → 15}}, PlotStyle → Thickness[0.016]]
```

Out[∘]=

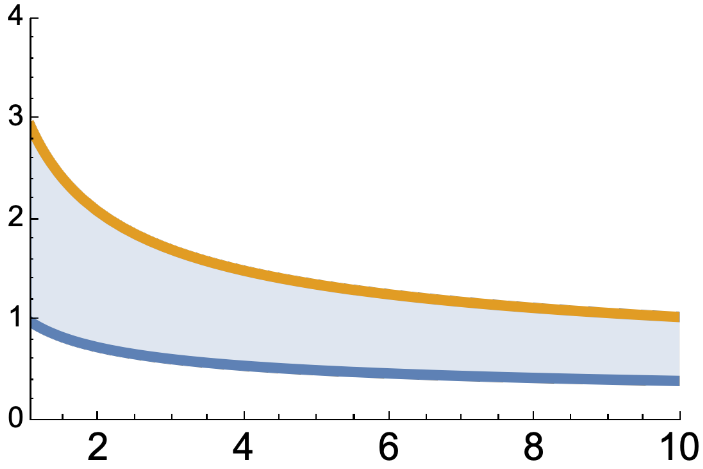

```
(*Fig. 3 *)
Clear [R, z, c]
Plot[{(Exp[z] - 1) (z + 1)/(R - 1) /. FindRoot[z + 1 == R (Log[R] - z)/(R - Exp[z]), {z, 0.5`}],
  -(e^-z (e^z - R))/(-1 + R) /. FindRoot[{e^(-2 z) (-1 + e^z) R == z}, {z, Log[R]/2}],
  (-e^z + R)/(-1 + R) /. FindRoot[{e^(-2 z) (-1 + e^z) R == z}, {z, Log[R]/2}]}, {R, 1, 20}, PlotStyle ->
  Directive[Hue[0.67`, 0.6`, 0.6`], Opacity[1.`], AbsoluteThickness[3.3`]],
 PlotRange -> {{1, 20}, {0, 0.7}}, TicksStyle -> {{FontSize -> 20}, {FontSize -> 15}},
 Ticks -> {{1, 5, 10, 15, 20}, {0, 0.1, 0.2, 0.3, 0.4, 0.5, 0.6, 0.7}}]
```

Out[ ]=

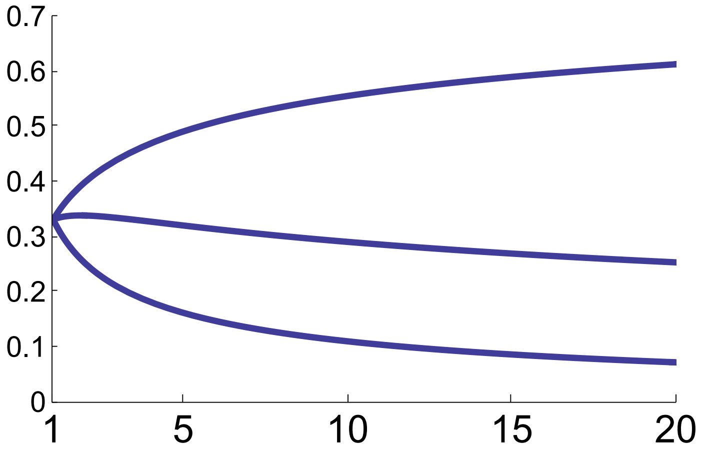

In[ ]:=
```
(*Fig. 4: Refuge *)
Plot[{1 / R, mu /. FindRoot[(1 - R)/(R (-1 + mu R)) == Log[((-1 + mu) R)/(-1 + mu R)], {mu, 1 / R / 2}]},
 {R, 1, 10}, PlotRange → {{1, 10}, {0, 1}}, Filling → {1 → {2}},
 TicksStyle → {{FontSize → 20}, {FontSize → 15}}, PlotStyle → Thickness[0.01]]
```

Out[ ]=

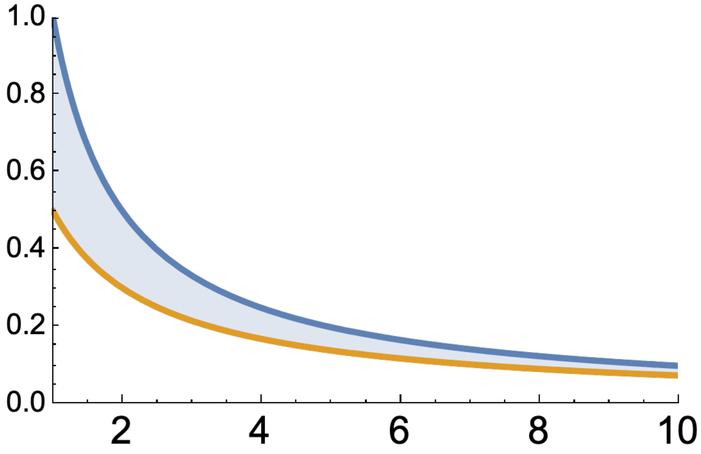

Supplement: S1 File — Wolfram Mathematica code used for generating the stability regions shown in Figs 1 and 2, and the limit of host suppression in Fig 3. (PDF) [file pone.0295980.s001.pdf]
