# Supplementary material for: Fundamental limits of parasitoid-driven host population suppression: Implications for biological control
Source: PLoS One. 2023 Dec 22;18(12):e0295980. doi: 10.1371/journal.pone.0295980 (PMC10745201; doi:10.1371/journal.pone.0295980)
Supplement: Attachment — Submitted filename: response.pdf [file pone.0295980.s002.pdf]

RESPONSE TO REVIEWERS

REVIEWER #1

> *The study aimed to model temporal dynamics considering trophic interactions, focusing on the suppression limits of hosts (pests) by parasitoids. In the presented formulation, self-limitation in host growth served as a resource for population regulation in the absence of parasitoids, characterizing a density-dependent system. Different scenarios were explored for parasitism occurring at different times: before, during, and after the host species growth limitation. The models also included a fraction of hosts protected from parasitism, establishing a refuge for hosts in the system.*
>
> *The results indicated that hosts with rapid growth, with parasitism occurring after host limited growth, exhibit the lowest pest density, conditioned by stable host-parasitoid coexistence. Regarding host refuge, the results suggested that specific reproduction rates lead to a critical value of protected hosts. The system's dynamics also remain stable for high parasitism rates, maintaining the host population at low sizes.*
>
> *The subject is crucial for theoretical ecology and provides a foundation for implementing biological pest control programs. The theoretical basis for the model is consistent and guides the reader in understanding the system's algebraic structure. I believe the study's results have merit to be published in PlosOne. However, I would like to offer some suggestions to enhance text clarity for readers*

**Response:** We thank the reviewer for reading and providing valuable feedback that has definitely improved the manuscript. We describe in further detail the changes made that are highlighted in red in the marked version of the paper.

> *Firstly, consider providing background information in the introduction to familiarize readers with the subject, particularly regarding suppression limits of hosts. Given the study's objective is to investigate host density suppression limits with parasitoids, it is important to introduce basic information about the parasitoid-host relationship in the context of pests, taking into account the abilities of hosts to escape from parasitoids and the challenges of parasitoids to reach hosts in an optimal way. Maybe a rapid paragraph explaining the possible consequences of too low or high parasitism rates for the equilibrium between species, mainly when parasitoids are not generalists, and need therefore moderately act on hosts to avoid subsequently its own exclusion from system. The current introduction starts by discussing the synchrony of life stages and introduces the mathematical model without explaining the study's relevance. Also, discussing the "host refuge" concept in the introduction could create opportune expectations for readers about its importance.*

**Response:** This a great suggestion and we have now modified the start of the introduction to present the fundamental question to be investigated and highlight the tradeoffs with parasitism rates. Changes have also been made below Eq. 3 to introduce the concept of host refuge in more detail.

> *While the authors mentioned plan to incorporate the Type II response in future studies, it is unclear why this issue was not addressed as clearly as the Type III response in the current manuscript. Also, regarding the first class of mechanisms, it is unclear why the parasitism escape function depended solely on parasitoid density, and not also the host density as explained in the second class of mechanisms.*

**Response:** We have rewritten this part of the introduction (please see below Eq. 3) where we now first introduce a Type II response and then discuss different stabilizing mechanisms providing more biological details on their origins. We have also now explicitly cited the modeling works related to each of these mechanisms that have detailed derivations of the escape responses showing its dependence on population densities.

> *The idea of a host refuge is intriguing, and the potential application scenarios mentioned are valuable. However, it might be worth considering whether the example involving parasitoid inaccessibility due to spatial location introduces the need to account for spatiality in the system. Although this point is touched upon at the end of the discussion concerning future implementations, addressing spatial location seems to leave a gap and could benefit from a referenced discussion showing real-world examples. Including more details about the examples, especially for non-technically familiar readers, could be beneficial.*

**Response:** We thank the reviewer for this suggestion and agree that providing a specific example of a host refuge would benefit the reader. We have modified the start of Section 6 providing the following example: "For example, the host *Bactrocera dorsalis* (oriental fruit fly) is parasitized by the pupal parasitoid *Dirhinus giffardii*. *B. dorsalis* larvae pupate below ground, and data shows that pupation depth determines the risk of parasitism – pupae further underground experience much lower rates of parasitism compared to pupae closer to the surface."

> *Regarding the example of the host's potential immune response to parasitism, supporting it with existing literature examples is equally relevant. Referencing literature examples would also bolster*

*the bases for inclusion of the refuge in the system since the results (Fig. 5) indicate significant dynamics differences for each scenario, affecting system stability.*

**Response:** We have cited references on host immune responses that form the key defense mechanism against attack by parasitoids.

*The caption of Figure 5 could provide more detail, identifying the curve colors and parameter 'c,' which presumably determines the parasitism rate (indicated by the downward arrow)*

**Response:** We apologize as there was a typo in Fig. 5. Parameter $c$ should have the parasitoid attack rate $c_p$. We have corrected this and explained it in the caption

*Lastly, approaching briefly in the article's discussion how the model could be adapted for scenarios beyond temperate regions, encompassing tropical areas with extensive plantations and high agricultural production would be valuable. This adaptation could offer critical insights for readers interested in applying the model to other scenarios, broadening the study's relevance for a wide-ranging readership.*

**Response:** Good point. We have now added this point at the end of the paper discussing model adaptation to tropical areas. More specifically we have added:

"Finally, it is important to point out that while here we have used the traditional discrete-time formalism of the Nicholson-Bailey model to investigate the limits of host density suppression, it will be interesting to extend these studies with the continuous-time framework of Lotka-Volterra that is more appropriate for modeling population dynamics of insects in the tropics."

REVIEWER #2

*The present work studies host-parasitoid interactions with a focus on biocontrol. The manuscript is well-written, with clear objectives, and robust results, showcasing the main points of the study. The Introduction is particularly strong, and the figures are well-presented. However, I believe the Discussion is somewhat shallow, especially in connecting the results with existing literature on biocontrol theory. While the references are extensive, their utilization could be more effective.*

**Response:** We thank the reviewer for the constructive comments and have strengthened the paper throughout as outlined in the responses for both referees.

> *"A typical life cycle is illustrated in Fig. 1 where adult hosts emerge during spring and lay eggs that hatch into larvae. Host larvae feed and grow on resources over summer, and then overwinter in the pupal stage to metamorphosize as adults the following year." Could you provide some examples of species that follow this specific life cycle? Certain moth species in temperate regions exhibit a different life cycle pattern from what you describe, such as the winter moth (Operophtera brumata) and the Bruce spanworm (Operophtera bruceata), in which they remain in the egg stage during winter and hatch into larvae in spring. Please specify which species or group of organisms the author is referring to, as life cycles can vary among different species.*

**Response:** The reviewer has correctly pointed out that life cycle patterns can vary a lot between species. Since these specifics of the host life cycle are not relevant to the model formulation and the results, we decided to simplify this part to make it less confusing for the readers. We have rewritten this to keep it as general as possible by removing the exact timing of when eggs hatch into larvae.

> *"A multitude of mechanisms are known to stabilize population dynamics and they can be classified into two types [11, 12]". The ecological significance of the two classes of mechanisms should be better explained. The first class includes the host refuge, which can be caused by some kind of protection, such as host defenses or host aggregation. The second class is caused by a Type III functional response, representing a form of population "refuge.".*

**Response:** Excellent suggestion. We have rewritten this part of the introduction (please see below Eq. 3) where we discuss the ecological significance of both these types of mechanisms. For completeness, we also added a few lines on Type II functional responses.

> *Furthermore, in the concluding remarks of your Discussion section, you reference several field studies that align with your results on the stabilizing effects of host refuges: "Our results coupling host refuge with the Beverton-Holt model reveal large regions of parameter space allowing stable coexistence (Fig. 4) consistent with field studies implicating proportional refuges in stabilizing host-parasitoid interactions [55-60]". It would be beneficial for readers if you could specify which of these studies pertain to the first class of mechanisms and which are related to the second. This distinction would provide a clearer context for understanding the ecological and biocontrol implications of your findings.*

**Response:** We have rewritten parts of the Introduction (see below Eq. (3)) emphasizing that proportional host refuges are in the first class of mechanisms. As per this, all these studies are directly related to the first class of mechanisms.

*I recommend the author to provide examples of pairs of species that exhibit these types of interactions. The CABI Digital Library is a valuable resource for gathering information about species with agricultural interest. Some starting examples are provided below, organized by the pest species and the corresponding parasitoids that attack different life stages.*

**Response:** We are particularly grateful for providing the names of pest species and their corresponding parasitoids. We have added one example of this in the Discussion and also provided an example of a pupal parasitoid at the start of Section 6 in the context of host refuge.

*Could you specify the software, methodology, and programming language employed for the two-parameter bifurcation diagrams? Ensuring that the results can be reproduced by independent researchers is essential.*

**Response:** This information has been added above Eq. 23. More specifically, we mention "The plot in Fig. 1 showing the stablity region was generated in Wolfram Mathematica, where for a given parameter set, $c_p^*$ was obtained by numerically solving (22). The population density trajectories were plotted in Microsoft Excel by iteratively solving the discrete-time model (18)."

*A deeper exploration of the implications of biocontrol would enrich the discussion. The primary goal of any biocontrol strategy is to reduce and control pest populations, and this aspect is not well addressed in the current manuscript. Additionally, an exploration of how the Allee effects might impact your results could enhance the manuscript. Drawing a parallel with the findings of Bompard et al. (2013) and Livadiotis et al. (2015), which you have cited (refs. [7] and [54], respectivelly), might provide valuable insights into the broader ecological context of your study.*

**Response:** This is a great suggestion and we have added this to the discussion. We discuss the primary goal of biocontrol and motivate the usage of models to determine the limits of host density suppression (second paragraph in Discussion) and discuss the inclusion of Allee effects (last paragraph in Discussion).

*Minor issues.*

**Response:** Thank you for finding these typos and they have been fixed in the revised version.
