# Supplementary material for: Fundamental limits of parasitoid-driven host population suppression: Implications for biological control
Source: PLoS One. 2023 Dec 22;18(12):e0295980. doi: 10.1371/journal.pone.0295980 (PMC10745201; doi:10.1371/journal.pone.0295980)
Supplement: Attachment — Submitted filename: response.pdf [file pone.0295980.s003.pdf]

RESPONSE TO REVIEWERS

REVIEWER #2

> *Following equation (4), the sentence "where Th is the handling time, and this has been shown to further destabilize the population dynamics" relates to the Discussion section on future research directions. "Future work will extend this study in several directions, such as incorporating a Type II functional response implemented using the semi-discrete approach". To prevent confusion, please clarify that the present study focuses on equation (3) with c_P constant. Although the Type II functional response is mentioned in the Introduction, it is not the focus of this study, which may cause misunderstandings. Similarly, the manuscript should explicitly indicate that it investigates only the first mechanism outlined in the paragraph following equation (4). This clarification is necessary because the Type III functional response, which is associated with the second class of mechanisms, is referenced but not examined in the study.*

Response: This a great suggestion. To clarify these points we have now added the following sentence just above Eq. 7

"In this contribution, we primarily focus on the case of a constant attack rate c_p (as in the Nicholson-Bailey model), and later in the manuscript we investigate the impact of proportional host refuges as outlined in the first class of mechanisms."

> *Typos and References:.*

Response: We thank the reviewer for meticulously reading the manuscript, and these typos in the text and references have been corrected.
